# Genome-Wide Analysis and Expression Profiles of *AhLOG* Gene Family in Peanut (*Arachis hypogaea* L.)

**DOI:** 10.3390/ijms27114958

**Published:** 2026-05-29

**Authors:** Boyang Zhang, Maoning Zhang, Guoquan Chen, Yue Wu, Pei Du, Suoyi Han, Tianlun Zhao, Liuyang Fu, Shuijin Zhu

**Affiliations:** 1 Hainan Institute, Zhejiang University, Sanya 572025, China; boyangz@zju.edu.cn (B.Z.); shjzhu@zju.edu.cn (S.Z.); 2 College of Agriculture and Biotechnology, Zhejiang University, Hangzhou 310058, China; 3 Key Laboratory of Oil Crops in Huang-Huai-Hai Plains, Ministry of Agriculture/Henan Provincial Key Laboratory for Oil Crops Improvement/National and Provincial Joint Engineering Laboratory for Peanut Genetic Improvement/The Shennong Laboratory/National Invocation Center for Bio-Breeding Industry, Institute of Crop Molecular Breeding, Henan Academy of Agricultural Sciences, Zhengzhou 450002, China; maoningzhang@126.com (M.Z.); dupei2005@163.com (P.D.); suoyi_han@126.com (S.H.); 4 School of Life Sciences, Zhengzhou University, Zhengzhou 450001, China; cgq2019zms@163.com (G.C.); wuyue99997@zzu.edu.cn (Y.W.)

**Keywords:** evolution, gene expression, LOG, peanut, stress tolerance

## Abstract

Peanut (*Arachis hypogaea* L.) is a globally vital oilseed and cash crop. The LONELY GUY (LOG) gene family acts as a core regulator of cytokinin activation, governing plant meristem maintenance, growth, development, and stress responses. However, the genome-wide characteristics, evolutionary dynamics, and biological functions remain largely uncharacterized in peanut. In this study, 24 *AhLOG* genes were identified from the cultivated peanut Tifrunner. Phylogenetic analysis, gene structure characterization, and conserved motifs validated the high evolutionary conservation of the *AhLOG* gene family, and subcellular localization prediction indicated most AhLOG proteins were distributed in the cytoplasm. Promoter cis-element analysis revealed abundant hormone-responsive and stress-responsive cis-elements in the promoter regions of the *AhLOG* genes. Synteny analysis uncovered highly conserved collinear relationships between cultivated peanut and its diploid progenitors (*A. duranensis*, *A. ipaensis*) as well as the wild tetraploid relative (*A. monticola*), while numerous conserved orthologous syntenic pairs were detected between peanut and the model plant *Arabidopsis thaliana*. Tissue expression profiles revealed remarkable functional divergence among members: *AhLOG3* and *AhLOG16* were widely involved in both vegetative and reproductive development, while several other *AhLOG* genes exhibited strict tissue-specific expression. Furthermore, qRT-PCR analysis demonstrated that *AhLOG* genes were significantly induced by abscisic acid (ABA), gibberellin (GA), indole-3-acetic acid (IAA), methyl jasmonate (MeJA), drought and salt treatments, with distinct expression patterns under these abiotic stress conditions. Collectively, this work provides a systematic understanding of the *AhLOG* gene family and offers key candidate genes along with theoretical support for further functional investigation and molecular breeding of stress-resistant peanut.

## 1. Introduction

Abiotic stress represents a major constraint on crop growth and productivity worldwide [1]. It has been estimated that abiotic stresses reduce average crop yields by more than 60% relative to their theoretical potential [2]. With ongoing global climate change and environmental deterioration, the adverse impacts of drought, waterlogging, salinity, and extreme temperatures on agricultural production have intensified dramatically [3,4,5,6], posing a severe threat to global food security and sustainable agricultural development [7]. To cope with fluctuating and harsh environmental conditions, plants have evolved elaborate regulatory networks during long-term adaptation, including phytohormone signal crosstalk, transcriptional regulation of stress-responsive genes, and biosynthesis of osmoprotective compounds [3,8]. Plant hormones act as central signaling molecules governing growth, development, and stress responses. The acquisition of abiotic stress tolerance relies on intricate mechanisms of stress perception, signal propagation, and downstream defense activation [9]. Upon exposure to adverse environments, abscisic acid (ABA), gibberellin (GA), indole-3-acetic acid (IAA), and methyl jasmonate (MeJA), and other hormones rapidly trigger cognate signaling cascades, establishing an integrated defense system from rapid stress responses to long-term acclimation [3,10,11].

As pivotal stress-responsive phytohormones, ABA, GA, IAA, and MeJA orchestrate adaptive growth and stress resistance via extensive crosstalk. Abscisic acid (ABA) confers rapid physiological stress tolerance by binding to its receptors to modulate stomatal movement and promote the accumulation of proline, soluble sugars and other osmoprotectants [12,13,14,15,16]. Gibberellins (GAs), key regulators of plant growth and development, generally antagonize ABA to balance growth and stress resistance, and their crosstalk fine-tunes resource allocation under adverse conditions [17,18,19,20,21,22,23,24,25,26,27]. Indole-3-acetic acid (IAA) mediates stress adaptation by regulating root architecture, reactive oxygen species (ROS) homeostasis and phytohormonal crosstalk [28,29,30,31,32,33,34]. Methyl jasmonate (MeJA) enhances stress tolerance by activating antioxidant systems, maintaining osmotic balance and coordinating hormonal interactions [35,36,37,38].

Previous studies have demonstrated that LONELY GUY (*LOG*) genes encode specific phosphoribohydrolases responsible for the direct activation of cytokinins, playing an essential role in cytokinin biosynthesis [39]. The *LOG* gene was first identified in rice through screening of mutants defective in meristem maintenance, and the one-step cytokinin activation pathway catalyzed by OsLOG was subsequently characterized [40]. As a cytokinin nucleoside 5′-monophosphate phosphohydrolase, OsLOG cleaves the linkage between the N^6^-substituted base and ribose 5′-monophosphate in cytokinin precursors such as trans-Zeatin Riboside-5′-monophosphate (iPRMP) and N^6^-(Δ^2^-Isopentenyl)adenosine-5′-monophosphate (tZRMP), converting cytokinin nucleotides into biologically active free-base forms. To date, *LOG* family members have been functionally characterized in *Arabidopsis thaliana* [41], rice [40], wheat [39] and cotton [42], and emerging evidence indicates that *LOG* genes participate in plant adaptation to drought, salinity, and other abiotic stresses by modulating CK homeostasis [43,44]. CKs themselves play crucial roles in balancing plant growth and stress resistance, regulating stomatal movement, antioxidant capacity, and osmotic balance under adverse conditions [44], further highlighting the significance of LOG-mediated CK activation in stress adaptation.

Peanut (*Arachis hypogaea* L.), an annual herbaceous legume native to tropical and subtropical regions of South America, is a globally important oilseed and cash crop [43]. Throughout its growth cycle, peanut is highly susceptible to multiple abiotic stresses, particularly drought, low temperature, and salinity. These stresses severely disrupt seed germination, flowering, pod development, nutrient accumulation, and other critical developmental processes, resulting in substantial yield losses and quality deterioration [44,45,46]. In recent years, considerable molecular research has focused on transcription factor families such as APETALA2/Ethylene-Responsive Factor (AP2/ERF), basic leucine zipper (bZIP), and WRKY in peanut [47,48,49]. However, the *LOG* gene family—a core regulator of cytokinin activation—has not been systematically identified or functionally characterized in cultivated peanut at the genome-wide level. Given the importance of *LOG* genes in CK biosynthesis and stress responses, a comprehensive analysis of the peanut *LOG* family is urgently needed to fill this research gap.

In this study, genome-wide identification was performed to characterize members of the *LOG* gene family in peanut, and comprehensive bioinformatic analyses were conducted to reveal their molecular characteristics. Transcriptomic profiling and quantitative real-time PCR (qRT-PCR) were employed to determine the expression patterns of *AhLOG* genes under abiotic stress conditions. The findings clarify the regulatory roles of the *AhLOG* gene family in peanut responses to abiotic stresses, establish a theoretical framework for dissecting cytokinin-mediated stress tolerance mechanisms in peanut, and provide valuable candidate gene resources for the genetic improvement and molecular breeding of stress-tolerant peanut cultivars.

## 2. Results

### 2.1. Protein Physicochemical Property Analysis and Identification of the AhLOG Gene Family

**Figure 1:**
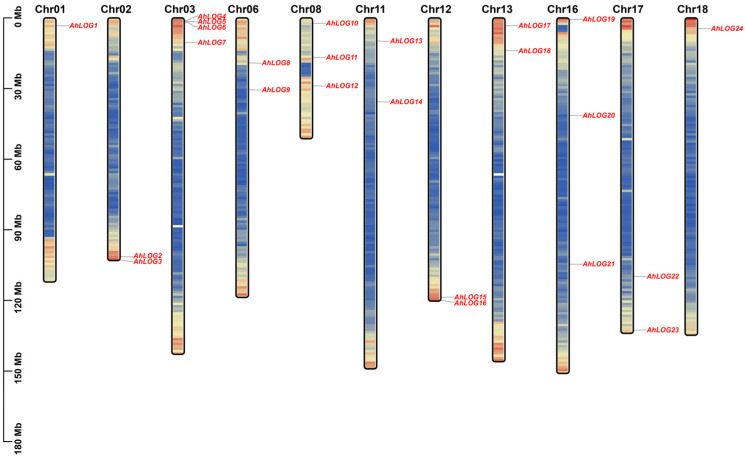
The chromosome mapping of *AhLOG* genes in the *Arachis hypogaea* genome. The physical locations of 24 *AhLOG* genes were mapped onto 11 chromosomes of *A. hypogaea* cv. Tifrunner. Chromosomes are labeled with their respective names and shown in vertical orientation, with a color gradient from red (chromosomal ends, high gene density) to deep blue (centromeric regions, low gene density). *AhLOG* gene names are indicated in red, and their positions along the chromosomes are marked accordingly. Most genes are located near chromosomal ends, with a conserved distribution pattern observed between homologous chromosomes of the A and B subgenomes.

A total of 24 *AhLOGs* were identified in the peanut (*Arachis hypogaea* L., cv. Tifrunner) genome by HMM search employing the Pfam domain PF03641 (the conserved LOG domain), followed by redundancy removal and conserved domain validation using the SMART v9.0 tool. These genes were designated sequentially as *AhLOG1* to *AhLOG24* based on their chromosomal physical locations (Table 1). The protein sequences of the *AhLOG* gene family members used in this study are listed in Supplementary Table S1. Among them, Chr03 harbored the largest number of *AhLOG* genes, with a total of 4 members. Chr08 and Chr16 each contained 3 genes, while Chr06, Chr11, Chr12, Chr13, and Chr17 each carried 2 genes. No *AhLOG* genes were detected on Chr04, Chr05, Chr07, Chr09, Chr10, Chr14, Chr15, Chr19, or Chr20 (Figure 1).

Protein physicochemical characterization revealed that peanut AhLOG proteins varied in length from 91 amino acids (aa; AhLOG6) to 316 aa (AhLOG1). The predicted molecular weights (MW) of the 24 AhLOG proteins ranged from 10.13 kDa (AhLOG6) to 35.41 kDa (AhLOG1), and their theoretical isoelectric points (*pI*) spanned from 5.07 (AhLOG17) to 8.85 (AhLOG21). The variations in MW and *pI* were primarily attributed to differences in amino acid composition (e.g., the contents of acidic and basic amino acids) and potential post-translational modifications (e.g., phosphorylation).

Subcellular localization predicted by WoLF PSORT demonstrated diverse distribution patterns of peanut AhLOG proteins across cellular compartments. In total, the 24 AhLOG proteins were distributed among four distinct subcellular compartments: 20 were localized in the Cytoplasm (83.33%), 2 in the Chloroplast, 1 in the Endoplasmic reticulum, and 1 in the Nucleus.

### 2.2. AhLOGs Phylogenetic Tree Analysis

**Figure 2:**
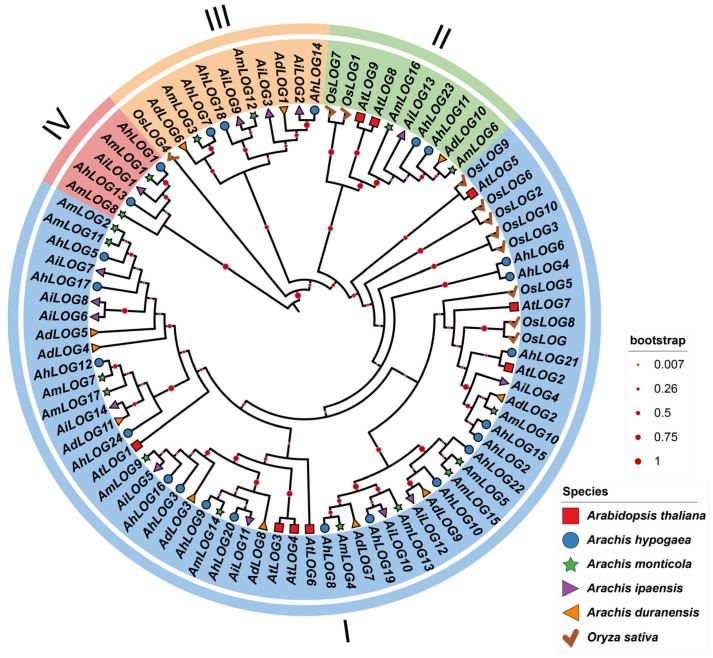
Phylogenetic tree of LOG proteins from peanut and model plants. Maximum Likelihood (ML) phylogenetic tree of the *LOG* gene family across cultivated peanut (*Arachis hypogaea*, *AhLOGs*), its wild relatives (*A. monticola*, *AmLOGs*; *A. ipaensis*, *AiLOGs*; *A. duranensis*, *AdLOGs*), and model plants, including *Arabidopsis thaliana* (*AtLOGs*) and *Oryza sativa* (*OsLOGs*). Roman numerals I, II, III, and IV represent four distinct subfamilies (Subfamily I–IV) classified according to the tree topology. Bootstrap analysis was performed with 1000 replicates to assess branch support. Different clades are distinguished by different colors. The scale bar indicates the number of amino acid substitutions per site, and the color bar represents bootstrap confidence values ranging from 0.5 to 1.0.

To investigate the phylogenetic relationships of *AhLOG* genes, a maximum likelihood (ML) phylogenetic tree was constructed using LOG protein sequences from cultivated peanut (*Arachis hypogaea*, AhLOGs), its wild relatives (*A. monticola*, AmLOGs; *A. ipaensis*, AiLOGs; *A. duranensis*, AdLOGs), and model plants, including *Arabidopsis thaliana* (AtLOGs) and *Oryza sativa* (OsLOGs) (Figure 2). Bootstrap analysis with 1000 replicates was performed to assess node support (threshold ≥ 0.7 for robust confidence). Based on the topological structure of the phylogenetic tree, all LOG proteins were tentatively grouped into four subfamilies (Subfamily I–IV). LOG proteins from different genera showed potential genus-specific clustering tendencies, while fine-scale relationships remained inconclusive due to low bootstrap support for some internal nodes. Notably, AhLOG1 and AhLOG13 from cultivated peanut were tightly clustered into an independent clade together with AiLOG1 (from diploid wild species *A. ipaensis*) and AmLOG1, AmLOG8 (from tetraploid wild species *A. monticola*), and this clade was located in Subfamily IV, which did not cluster with any LOG members from model plants *A. thaliana* and *O. sativa*, indicating that this clade represents a unique evolutionary lineage specific to *Arachis* species. These results suggest the high evolutionary conservation of the LOG gene family across plant lineages. Cultivated wild *Arachis* LOGs were interspersed without distinct species-specific clades, reflecting the conservation during peanut domestication. Additionally, *Arachis* species harbored more LOG genes than *A. thaliana* and *O. sativa*, indicating lineage-specific expansion of the *AhLOG* family under the background of ancient evolutionary conservation.

### 2.3. Analysis of AhLOGs Protein Conserved Domain and Structure

**Figure 3:**
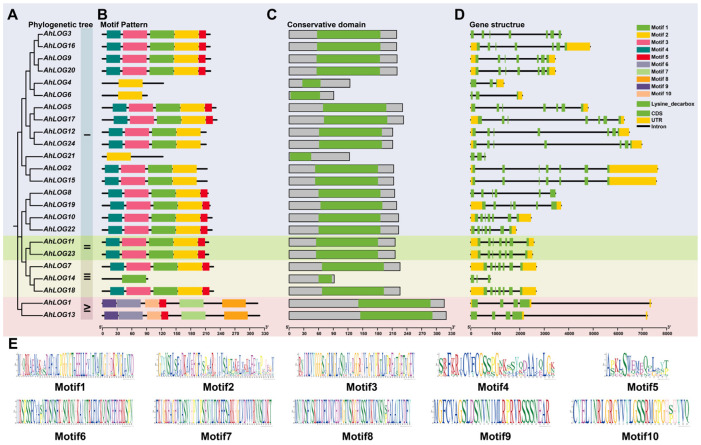
Conserved motifs, domains and gene structures of *AhLOG* genes in peanut. (**A**) Phylogenetic tree of the *AhLOG* gene family. (**B**) Distribution of 10 conserved motifs in AhLOG proteins identified by MEME. Different motifs are labeled with colored boxes and numbered 1–10. (**C**) Conserved domain analysis showing the LOG domain (Lysine_decarbox, PF03641) within AhLOG proteins. (**D**) Exon–intron structure of *AhLOG* genes. Yellow rectangles represent exons, light green rectangles represent untranslated regions (UTRs), and gray lines connecting exons represent introns. (**E**) Alignment of the 10 conserved motifs (Motif 1–10) generated by the MEME online website. The sequence logo for each motif shows the amino acid conservation pattern, with different colors representing distinct physicochemical properties of amino acids.

Using MEME conserved motif analysis, ten conserved motifs were identified among members of the LOG gene family (Table S2), which were designated as Motif 1 to Motif 10 in sequence. The core conserved domain of the LOG family, Lysine_decarbox (PF03641), was mainly enriched in Motif 1, Motif 2, Motif 3, Motif 5, Motif 6, and Motif 7, which constituted the functional core region of the family. The distribution patterns of conserved motifs among phylogenetic subgroups were highly consistent with gene structure characteristics (Figure 3). Most conserved motifs were widely present in nearly all *AhLOG* members, and only a few motifs exhibited branch-specific distribution. For instance, members of certain evolutionary branches specifically contained Motif 6 and Motif 9, which was highly consistent with their phylogenetic clustering results; in contrast, *AhLOG4*, *AhLOG6*, and *AhLOG21* only harbored the core Motif 2, and *AhLOG14* contained only one core Motif 1, reflecting slight structural differentiation within the family. Collectively, the vast majority of conserved motifs were stably present in the *AhLOGs* (Figure 3A). Combined analyses of phylogenetic relationships, conserved motif patterns and gene structures confirmed the high consistency of gene organizations within the same subgroup, indicating that the amino acid sequences of *AhLOGs* are highly conserved, and members within the same evolutionary clade may perform similar biological functions.

Divergences in exon–intron architecture and amino acid substitutions can cause variations in coding regions, thereby potentially altering gene function. To dissect the variation rules of gene structure in the *AhLOG* gene family, we examined the exon–intron arrangement characteristics of these genes. The results showed that closely related members within the same phylogenetic clade generally exhibited comparable exon–intron structures. For gene structure analysis, the exon counts of *AhLOG* genes varied from 3 to 7, and the intron counts ranged from 2 to 6. Notably, the majority of *AhLOG* genes presented the canonical genomic structure consisting of 7 exons and 6 introns, and these dominant genotypes displayed highly conserved exon–intron architectures. Overall, genes within the same subgroup displayed similar structures, with structural differences detected in only a few members (Figure 3). Among all analyzed members, only *AhLOG3*, *AhLOG14*, and *AhLOG21* lacked UTRs, while the remaining members harbored UTR structures; intron reduction was observed in extremely few genes, suggesting that exon gain or loss events rarely occurred during the evolution of the *LOG* gene family. In addition, gene structures of members within specific subgroups were highly matched with their phylogenetic clustering relationships, further verifying the evolutionary conservation of gene structures in this family.

### 2.4. Predicted Cis-Regulatory Elements in the Promoter Regions of AhLOG Genes

**Figure 4:**
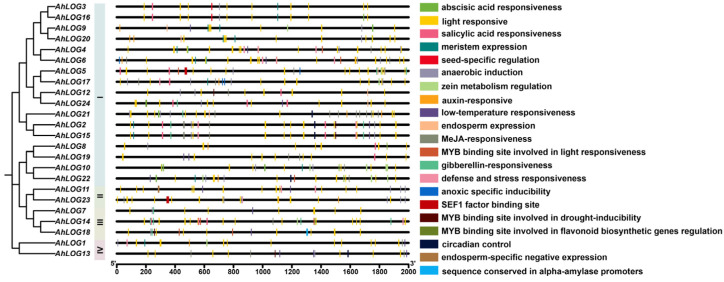
Analysis of predicted cis-regulatory elements in the promoters of *AhLOG* genes. Different colors indicate distinct functional categories: light-responsive (Box4, GATA-motif, GT1-motif, I-box), hormone-responsive (ABRE, TCA, MeJA, auxin, GA), stress-responsive (LTR, defense, anaerobic, anoxic, drought-inducible), and development-related (meristem, seed, endosperm, zein metabolism, circadian control). The Roman numerals I–IV represent the four phylogenetic groups of *AhLOG* genes.

To gain insights into the function of *AhLOGs* in peanut development, growth, and responses to abiotic stressors and plant hormone treatment, the cis-regulatory components in *AhLOG* promoters were examined (Table S3). We focused on three major categories of cis-regulatory elements: phytohormone responsiveness, abiotic stress responsiveness, and development and growth regulation (Figure 4). Among abiotic stress response elements, light-responsive elements were detected as the most abundant type, followed by low-temperature responsiveness, defense and stress responsiveness, anaerobic induction, and anoxic specific inducibility elements. Diverse light-responsive motifs (including GT1-motif, I-box, and Box 4) accounted for the largest proportion of abiotic stress-related elements, indicating that light signals serve as a key regulatory factor for *AhLOG* gene transcription. In addition, low-temperature responsive (LTR) elements, defense and stress responsive elements, as well as anaerobic/anoxic specific inducibility elements were widely distributed, suggesting that *AhLOG* genes participate in peanut adaptation to multiple abiotic stress environments.

Furthermore, we also identified six types of cis-elements related to development and growth in *AhLOG* promoters, which are involved in regulating meristem expression, seed-specific regulation, endosperm expression, endosperm-specific negative expression, zein metabolism regulation, and circadian control. Among them, meristem expression and endosperm expression elements were relatively abundant, revealing that *AhLOG* genes are actively implicated in peanut meristem differentiation, seed and endosperm development, and nutrient metabolism processes. Meanwhile, circadian control elements suggested that partial *AhLOG* genes are regulated by circadian rhythms.

These bioinformatic predictions suggest that certain cis-elements are broadly and distributed across most *AhLOG* promoters, while others exhibit gene-specific distribution patterns. Based on these predicted elements, it is plausible that the expression patterns of *AhLOGs* may vary across different stages of peanut development and may be influenced by phytohormone signals and abiotic stress conditions Notably, these are only bioinformatic predictions whose regulatory functions need experimental validation.

### 2.5. Tandem Duplication, and Selection Pressure Analysis of AhLOGs

**Figure 5:**
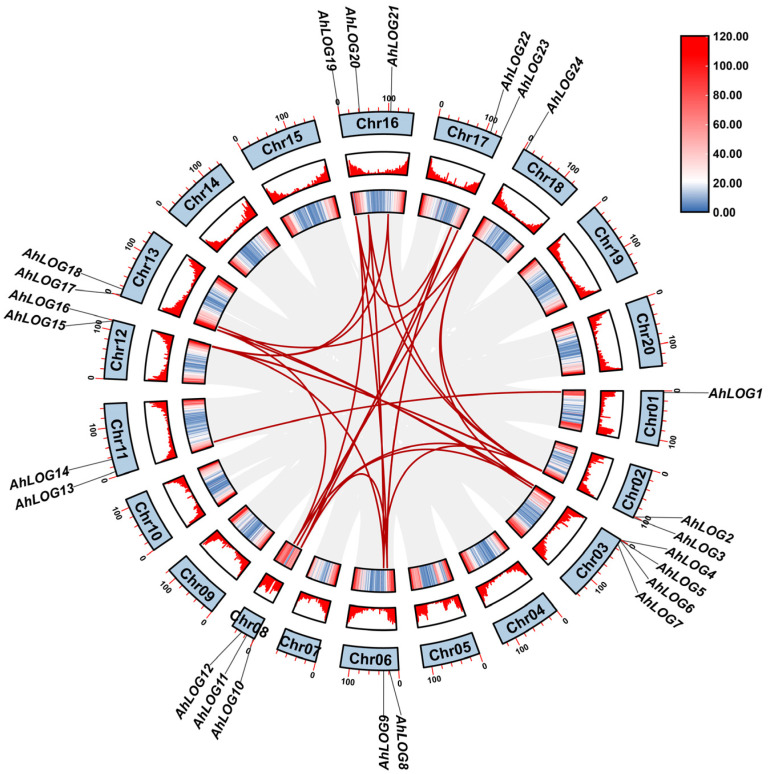
Intraspecific syntenic relationships of *AhLOG* genes in the *Arachis hypogaea* genome. Circular representation of syntenic relationships among 24 *AhLOG* genes across the 20 chromosomes of *A. hypogaea* (AABB genome). Gene locations are labeled on the outer ring, with red histograms indicating gene density. Colored connecting lines within the circle denote 28 segmental duplication pairs identified among *AhLOG* genes. Notably, all *AhLOG* members except *AhLOG14* are involved in segmental duplications, and no tandem duplication events are detected in the *AhLOG* gene family.

Tandem and whole-genome duplications are crucial processes that drive the expansion of gene families, enhance genome complexity, and promote evolutionary innovation. By analyzing the chromosomal distribution of 24 identified *AhLOG* genes, we found that these genes are unevenly distributed across 11 chromosomes of cultivated peanut, and no *AhLOG* genes were detected on the remaining chromosomes (Table 1). Through the analysis of gene duplication events, a total of 28 intragenomic collinear gene pairs derived from segmental duplication were identified (Figure 5). Notably, all *AhLOG* members except *AhLOG14* were involved in segmental duplication events, while no tandem duplication events were detected in the *AhLOG* gene family. These results demonstrated that segmental duplication is the primary driving force underlying the expansion and evolution of the peanut *LOG* gene family.

The non-synonymous substitution rate (Ka) reflects the rate of mutations that alter amino acids, while the synonymous substitution rate (Ks) represents the rate of base substitutions that do not alter amino acids; together, these two parameters are used to infer the frequency of gene duplication events and the divergence time of duplicated pairs. To estimate the evolutionary rate of duplication events, infer the divergence time of duplicated gene pairs, and investigate the duplication history and evolutionary constraints acting on the *AhLOG* gene family in *Arachis hypogaea* (peanut), we calculated Ka and Ks values for 28 valid *AhLOG* gene pairs. Notably, *AhLOG14* was excluded from the analysis as no collinear partner was detected (Table 2). No tandem duplication pairs were analyzed, as none were identified in the *AhLOG* gene family. Among the 28 valid gene pairs, 8 pairs exhibited low Ks values ranging from 0.0068 to 0.0408, indicating recent duplication events. Using a molecular clock calibration of t = Ks × 61.5 Mya (where t represents divergence time), these duplications are estimated to have occurred approximately 0.42 to 2.51 million years ago (Mya). The remaining 20 pairs showed substantially higher Ks values (0.4529–1.1274), corresponding to older segmental duplication events with divergence times ranging from 27.9 to 69.4 Mya. All 28 duplicated pairs had Ka/Ks ratios below 1 (ranging from 0 to 0.7837). This result suggests that the *AhLOG* gene family has evolved primarily under purifying selection—this selection pressure has strongly preserved the ancestral functions of the proteins encoded by *AhLOG* genes (Table 2).

### 2.6. Collinearity Analysis of LOG Genes in Arachis and Model Plants

**Figure 6:**
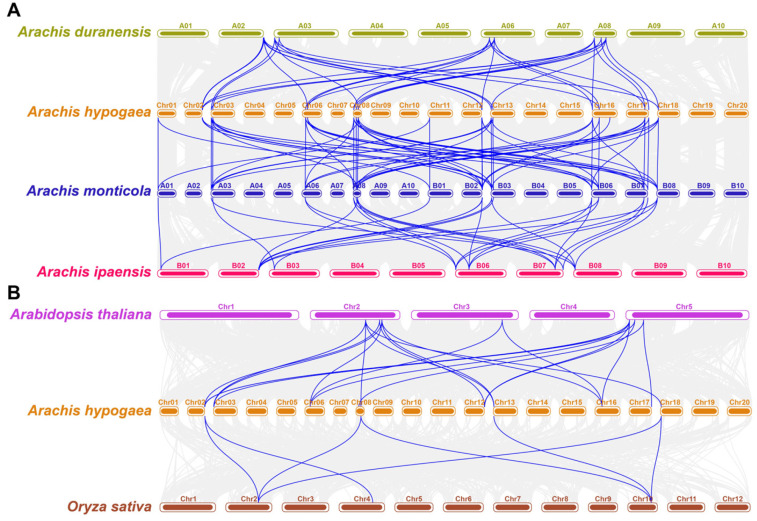
Interspecific syntenic relationships of *AhLOG* genes between *Arachis hypogaea* and other plant species. Blue lines indicate conserved syntenic gene pairs. (**A**) Collinearity between cultivated peanut (*A. hypogaea*, AABB) and its wild relatives: the diploid progenitors *A. duranensis* (AA genome) and *A. ipaensis* (BB genome), as well as the tetraploid wild species *A. monticola* (AABB). Extensive syntenic pairs are observed, supporting the allotetraploid origin of cultivated peanut and indicating near-complete syntenic conservation of LOG genes during polyploidization. (**B**) Collinearity of the *AhLOG* gene family between *A. hypogaea* and model plants *Arabidopsis thaliana* (dicot) and *Oryza sativa* (monocot). More conserved syntenic pairs were identified between peanut and *Arabidopsis* than between peanut and rice, confirming the deep evolutionary conservation of *LOG* genes across angiosperms.

To elucidate the evolutionary conservation and phylogenetic relationships of the *LOG* gene family among *Arachis* species, interspecific synteny analyses were performed among cultivated peanut (*Arachis hypogaea*, AABB), its diploid wild progenitors (*A. duranensis*, AA; *A. ipaensis*, BB), and the wild tetraploid relative (*A. monticola*, AABB) (Figure 6A). Further collinearity analyses revealed extensive syntenic pairs between *LOG* genes of cultivated peanut (*AhLOG*) and those of its wild relatives, including *AdLOG* (*A. duranensis*), *AiLOG* (*A. ipaensis*), and *AmLOG* (*A. monticola*). For instance, multiple *AhLOG* genes (e.g., *AhLOG2*, *AhLOG3*, *AhLOG5*, *AhLOG8*, *AhLOG12*, and *AhLOG15–24*) matched multiple syntenic *AdLOG* genes on chromosomes A02, A03, A06, and A08 of *A. duranensis*. Similarly, dense syntenic blocks were observed between *AhLOG* and *AmLOG*, which were distributed across multiple chromosomes of *A. monticola* (e.g., A01, A03, A06, A08, Am11–B03, and B06–B08). Moreover, collinearity comparisons between *AmLOG* and *AiLOG* revealed clear orthologous correspondences between *AmLOG* genes and chromosomes B01–B03 and B06–B08 of the B subgenome in *A. ipaensis*. Collectively, these findings strongly support the allotetraploid origin model of cultivated peanut, in which the A subgenome was derived from *A. duranensis* and the B subgenome from *A. ipaensis*. Additionally, near-complete syntenic conservation of *LOG* genes was detected between the wild tetraploid *A. monticola* and cultivated peanut, indicating extensive structural conservation of the *LOG* gene family during the polyploidization of *Arachis* species.

To dissect the evolutionary conservation of the peanut *LOG* gene family, synteny analyses were performed between cultivated peanut and two model plants, *Arabidopsis thaliana* and *Oryza sativa* (Figure 6B). The results revealed 22 syntenic pairs between peanut *AhLOG* genes (including *AhLOG2* to *AhLOG6*, *AhLOG9*, *AhLOG11*, *AhLOG12*, *AhLOG15*, *AhLOG16*, *AhLOG17*, *AhLOG20*, *AhLOG23* and *AhLOG24*) and *Arabidopsis AtLOG* genes, which were mainly distributed on chromosomes Chr2, Chr3, and Chr5 of *A. thaliana*. Several *AhLOG* genes showed direct orthologous synteny with *AtLOG1*, *AtLOG3*, or *AtLOG6*. In contrast, only 7 syntenic pairs were identified between peanut and rice, involving *AhLOG3*, *AhLOG12*, *AhLOG17*, *AhLOG24*, and *OsLOG2*, *OsLOG6*, *OsLOG10*. The striking difference in syntenic pair number may result from lineage-specific expansion, genomic rearrangements after the divergence of monocots and dicots, or extensive gene loss and structural variations in the rice genome. Abundant syntenic orthologous pairs were identified between peanut and *Arabidopsis thaliana*, while only limited syntenic relationships were detected between peanut and monocot rice. Collectively, these findings support the ancient evolutionary origin of the *LOG* gene family, rather than widespread conserved synteny across all angiosperms.

### 2.7. Expression Patterns of AhLOG Genes in Peanut Tissues

**Figure 7:**
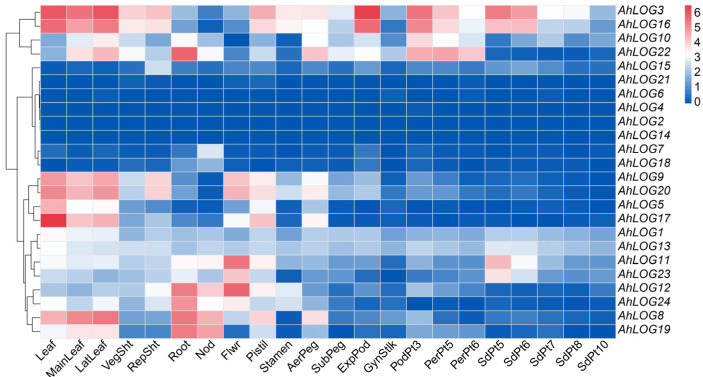
Expression profiles of *AhLOG* genes across different tissues, visualized by heatmaps. All heatmaps were generated based on FPKM values normalized by log_2_(x + 1) transformation. Red and blue colors indicate high and low gene expression levels, respectively. Hierarchical clustering was performed for genes (rows) but not for samples (columns) to preserve the original temporal and tissue order. Expression profiles of *AhLOG* genes across 22 tissues in *Arachis hypogaea* cv. Tifrunner. Tissue abbreviations: Leaf, main leaf (MainLeaf), lateral leaf (LatLeaf), vegetative shoot (VegSht), reproductive shoot (RepSht), root (Root), nodule (Nod), flower (Flwr), pistil (Pistil), stamen (Stamen), aerial peg (AerPeg), subterranean peg (SubPeg), expanding pod (ExpPod), gynophore (GynStlk), pod stage 3 (PodPt3), pericarp stage 5 (PerPt5), pericarp stage 6 (PerPt6), seed stage 5 (SdPt5), seed stage 6 (SdPt6), seed stage 7 (SdPt7), seed stage 8 (SdPt8), seed stage 10 (SdPt10).

To characterize tissue-specific expression of the *AhLOG* gene family in peanut, we analyzed transcriptome FPKM values of 24 *AhLOG* genes across 22 tissues/organs of Tifrunner. Based on expression profiles, genes were divided into three groups: five genes (*AhLOG2*, *AhLOG4*, *AhLOG6*, *AhLOG14*, *AhLOG21*) showed negligible expression (FPKM < 0.1) in all tissues (likely pseudogenes or stress-specific genes); several genes (e.g., *AhLOG1*, *AhLOG13*, *AhLOG15*) showed constitutive moderate expression for housekeeping roles; the rest exhibited obvious tissue specificity or high expression, indicating key functions in organ development (Figure 7). The *AhLOG* gene family exhibited distinct tissue-specific expression profiles in various tissues of peanut Tifrunner. *AhLOG22*, *AhLOG8*, *AhLOG12*, *AhLOG19*, and *AhLOG24* were highly expressed in roots, with *AhLOG19* showing specific high expression in nodules, implying its potential function in symbiotic nitrogen fixation. *AhLOG3* and *AhLOG17* exhibited extremely high transcript levels in leaves, and *AhLOG17* displayed an almost leaf-specific expression pattern, serving as a key marker for leaf development. *AhLOG11* and *AhLOG12* were abundantly and specifically expressed in floral organs, indicating their essential roles in flower development. Meanwhile, *AhLOG3* and *AhLOG16* showed extraordinarily high expression in expanding pods and early seeds, suggesting their core functions in pod enlargement and early seed development. Additionally, *AhLOG22* was highly expressed in roots, pegs, and pericarp, associated with the development of underground tissues. Collectively, the *AhLOG gene family* shows obvious functional divergence: *AhLOG3* and *AhLOG16* are widely involved in multiple developmental processes, while *AhLOG17*, *AhLOG11/12*, *AhLOG19*, and *AhLOG22* exhibit high tissue specificity to perform unique physiological functions.

### 2.8. Expression Profiles of AhLOGs in Response to ABA, GA, IAA, and MeJA Treatments by qRT-PCR

**Figure 8:**
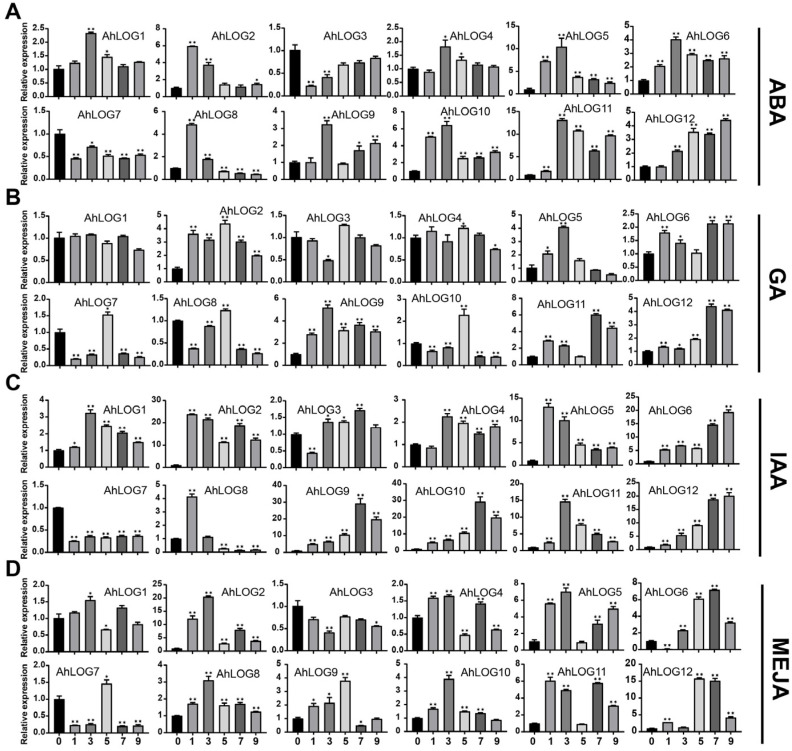
Expression profiles of *AhLOG* genes in response to exogenous phytohormone treatments. Quantitative reverse-transcriptase PCR (qRT-PCR) was used to analyze the relative expression levels of 12 *AhLOG* genes (*AhLOG1–12*) from the A subgenome in peanut seedlings treated with (**A**) 20 μM abscisic acid (ABA), (**B**) 100 μM gibberellin (GA), (**C**) 100 μM indole-3-acetic acid (IAA), and (**D**) 10 μM methyl jasmonate (MeJA). Samples were collected at 9 h post-treatment. Expression data were normalized against the internal reference gene *AhADH3*. Error bars indicate the standard deviation of three biological replicates, each with three technical replicates. The 0 h group served as the untreated control group. Stars indicate that gene expression levels at 1, 3, 5, 7, and 9 h showed significant differences compared to the 0 h time point after *t*-test (* *p* < 0.05, ** *p* < 0.01).

Phytohormones serve as core signaling molecules regulating plant growth, development, and abiotic stress responses, with extensive crosstalk between hormone and stress pathways. To determine the hormone-responsive expression patterns of *AhLOG* genes, peanut seedlings were treated with 20 μM ABA (Figure 8A), 100 μM GA (Figure 8B), 100 μM IAA (Figure 8C), and 10 μM MeJA (Figure 8D), respectively. Given the allotetraploid (AABB) nature of cultivated peanut and the high sequence similarity between A and B subgenomes, we specifically analyzed the expression of 12 *AhLOG* genes (*AhLOG1–12*) from the A subgenome by qRT-PCR post-treatment (Figure 8, Table S4). The results showed that nine *AhLOG* genes (*AhLOG1*, *AhLOG2*, *AhLOG5*, *AhLOG6*, *AhLOG8*, *AhLOG9*, *AhLOG10*, *AhLOG11*, and *AhLOG12*) were upregulated by ABA, while *AhLOG3* and *AhLOG7* were downregulated. Under GA treatment, six genes (*AhLOG2*, *AhLOG5*, *AhLOG6*, *AhLOG9*, *AhLOG1*1, and *AhLOG12*) were induced, and only *AhLOG1* was repressed. In response to IAA, 11 genes (*AhLOG1*, *AhLOG2*, *AhLOG3*, *AhLOG4*, *AhLOG5*, *AhLOG6*, *AhLOG8*, *AhLOG9*, *AhLOG10*, *AhLOG11*, and *AhLOG12*) showed increased expression, whereas *AhLOG7* was downregulated. Following the MeJA application, 10 genes (*AhLOG1*, *AhLOG2*, *AhLOG4*, *AhLOG5*, *AhLOG6*, *AhLOG8*, *AhLOG9*, *AhLOG10*, *AhLOG11*, and *AhLOG12*) were upregulated, and only *AhLOG3* exhibited decreased transcription. The primers used for the expression analysis are listed in Table S5.

### 2.9. Expression Profiles of AhLOGs Under Drought and Salt Stress by qRT-PCR

**Figure 9:**
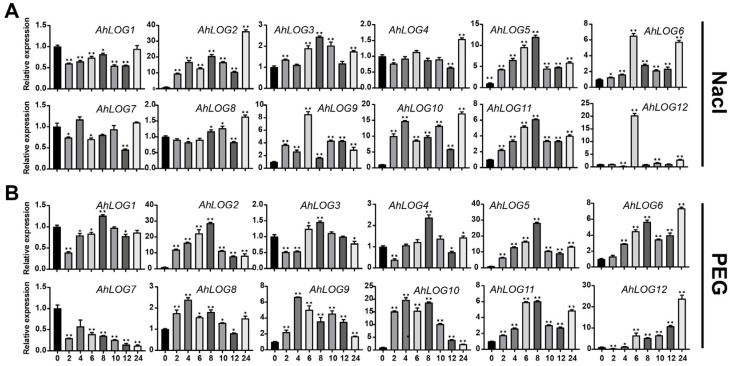
Expression profiles of *AhLOG* genes in response to drought and salt stresses in peanut. Quantitative reverse-transcriptase PCR (qRT-PCR) was used to analyze the relative expression levels of 12 *AhLOG* genes (*AhLOG1–12*) from the A subgenome in peanut seedlings subjected to (**A**) 350 mM PEG6000 (simulated drought stress) and (**B**) 200 mM NaCl (salt stress) for 24 h. Expression data were normalized against the internal reference gene *AhADH3*. Error bars indicate the standard deviation of three biological replicates, each with three technical replicates. The 0 h group served as the untreated control group. Stars indicate that gene expression levels at 2, 4, 6, 8, 10, 12 and 24 h showed significant differences compared to the 0 h time point after *t*-test (* *p* < 0.05, ** *p* < 0.01).

Drought and salt stress are major abiotic stresses restricting plant growth and development, with highly conserved regulatory mechanisms between the two stresses. To explore the response patterns of *AhLOG* genes to drought and salt stress, peanut seedlings were treated with 350 mM PEG6000 (simulated drought) (Figure 9A) and 200 mM NaCl (simulated salt) (Figure 9B) for 24 h, and gene expression levels were detected by qRT-PCR. Considering that cultivated peanut is an allotetraploid (AABB) with high sequence similarity between the A and B subgenomes, only 12 *AhLOG* genes (*AhLOG1*–*12*) located on the A subgenome were analyzed (Figure 9). The results showed that nine *AhLOG* genes, including *AhLOG2*, *AhLOG4*, *AhLOG5*, *AhLOG6*, *AhLOG8*, *AhLOG9*, *AhLOG10*, *AhLOG11*, and *AhLOG12*, were significantly upregulated under drought stress, while only *AhLOG7* was downregulated. Under salt stress, seven *AhLOG* genes (*AhLOG2*, *AhLOG3*, *AhLOG5*, *AhLOG6*, *AhLOG9*, *AhLOG10*, and *AhLOG11*) were upregulated. Comprehensive analysis revealed that six *AhLOG* genes (*AhLOG2*, *AhLOG5*, *AhLOG6*, *AhLOG9*, *AhLOG10*, and *AhLOG11*) were significantly induced by both drought and salt stress, suggesting that these genes might participate in the regulation of osmotic balance and stress signal transduction, and play dual roles in peanut tolerance to drought and salt stress.

## 3. Discussion

LONELY GUY (LOG) proteins serve as terminal catalytic enzymes in cytokinin activation and play critical roles in meristem maintenance, organogenesis, and stress adaptation in plants [39,41]. Accumulating evidence has verified the crucial roles of *LOG* family genes in abiotic stress tolerance across multiple plant species; for instance, *RcLOG5* enhances drought, salt, and cold resistance in transgenic *Arabidopsis* [50], while *GhLOG5* is implicated in cytokinin metabolism and drought tolerance in cotton [51], and several *OsLOG* genes in rice show significantly altered expression under drought and salt treatments [52]. However, the genome-wide characteristics of this family in cultivated peanut have long remained uncharacterized. In this study, we systematically identified the *AhLOG* gene family in peanut for the first time and found that it comprises 24 members, a number significantly larger than those in *Arabidopsis thaliana* and *Oryza sativa* [41,50], suggesting that this family has undergone lineage-specific expansion in peanut. Evolutionary dynamic analysis showed that segmental duplication (rather than tandem duplication) is the major mechanism driving this expansion, and all duplicated gene pairs are under purifying selection (Ka/Ks < 1). This selective pressure explains why the *AhLOG* gene family maintains high structural and functional conservation during allotetraploidization: collinearity analysis revealed extensive collinearity of *LOG* genes between cultivated peanut and its wild diploid peanut (*A. duranensis*, *A. ipaensis*) as well as the wild tetraploid peanut *A. monticola*, providing molecular evidence for the allotetraploid origin of peanut and indicating that the core functions of *LOG* are under strict constraint [40]. Despite overall conservation, moderate functional divergence still exists within the *AhLOG* gene family. Phylogenetic analysis revealed that LOG proteins from *Arachis* species form independent clades distinct from those of *Arabidopsis* and rice, among which a highly supported clade containing *AhLOG1/AhLOG13* and their orthologs from wild relatives is unique to *Arachis*, implying that this clade may have acquired lineage-specific novel functions. Meanwhile, a small number of *AhLOG* members (e.g., *AhLOG4*, *AhLOG6*, *AhLOG21*) retain only core motifs, whereas most members possess a complete set of conserved motifs and stable exon–intron structures (mostly 7 exons/6 introns). This structural feature of “core conservation with marginal variation” enables the family to achieve subfunctionalization or neofunctionalization through subtle sequence differences on the premise of maintaining basic catalytic functions, thereby adapting to the specific developmental requirements of peanut [40,41].

Although cis-element predictions are bioinformatic in nature and require experimental validation, they offer valuable hypotheses for regulatory function. The promoter regions of *AhLOG* genes are predicted to contain diverse cis-acting elements, with light-responsive elements being the most abundant. This observation raises the possibility that light signaling is the core upstream factor for their transcriptional regulation. In addition, elements related to abiotic stress (low temperature, anaerobic, defense), developmental processes (meristem, endosperm), and circadian rhythm are widely distributed, hinting at a potential multi-dimensional regulatory network [3,8]. It is important to note, however, that these predictions are based on silico scanning (PlantCARE, https://bioinformatics.psb.ugent.be/webtools/plantcare/html/, accessed on 4 April 2026), which is known to produce false-positive results; therefore, the proposed regulatory roles of individual cis-elements require direct experimental validation. Notably, this diversity in promoter structure does not lead to random expression patterns, but instead presents tissue specificity highly coupled with the biological characteristics of peanut. For example, *AhLOG3* and *AhLOG16* are extremely highly expressed in expanding pods and early seeds, consistent with the unique developmental processes of underground pod enlargement and seed setting in peanut; *AhLOG17* shows leaf-specific expression, while *AhLOG11/12* are enriched in floral organs, indicating their involvement in photosynthetic organ formation and reproductive development, respectively. Furthermore, multiple *AhLOG* genes highly expressed in roots/nodules (e.g., *AhLOG19*) may participate in symbiotic nitrogen fixation by regulating cytokinin homeostasis, a key trait for high nitrogen use efficiency in legume crops. Given the high false-positive rate of cis-element predictions from PlantCARE bioinformatic analysis, further experimental validation using promoter-reporter assays, deletion analysis, and electrophoretic mobility shift assay (EMSA) is required in future investigations.

Functional divergence is reflected not only in developmental regulation but also in stress responses. qRT-PCR results showed that most *AhLOG* genes can be significantly induced by abscisic acid (ABA), gibberellin (GA), auxin (IAA), and methyl jasmonate (MeJA), and this induction pattern is highly consistent with the hormone-responsive elements in their promoters. As core signaling molecules, plant hormones coordinate plant growth and stress resistance through complex crosstalk networks [3,10,11]: ABA confers rapid tolerance by inducing stomatal closure and accumulation of osmotic adjustment substances [12,13,14]; GA often antagonizes ABA to balance resource allocation [17,18,19,20,21]; IAA enhances stress adaptation by regulating root system architecture and reactive oxygen species (ROS) homeostasis [28,29,30,31]; MeJA improves stress tolerance by activating the antioxidant system [35]. More importantly, six genes, including *AhLOG2*, *AhLOG5*, *AhLOG6*, *AhLOG9*, *AhLOG10*, and *AhLOG11*, are simultaneously and significantly up-regulated by drought and salt stresses, indicating that they are shared nodes in response to osmotic and ionic stresses. Previous studies have shown that cytokinins can improve plant stress tolerance by regulating stomatal movement, antioxidant system, and osmotic balance [44]; therefore, these stress-induced *AhLOG* genes may exert protective functions by activating cytokinins. This characteristic of multiple responses to “development–hormone–stress” makes the *AhLOG* gene family a core regulatory module linking growth and stress resistance [3,8]. Notably, the high sequence homology between the A and B subgenomes of peanut poses challenges for designing subgenome-specific primers, limiting our qRT-PCR analysis to *AhLOG* genes from the A subgenome. This represents a limitation of the present study. The varying Ka/Ks ratios suggest potential regulatory divergence between the two subgenomes. Future work will focus on designing SNP-based subgenome-specific primers to complement and validate these findings. While our time-series expression profiling captured major stress-responsive patterns of *AhLOG* genes, the 1 h and 2 h sampling intervals for hormone and abiotic stress treatments may fail to detect ultra-transient early-stage or long-term late-phase regulatory responses. Further research adopting higher-density time point sampling and multi-omics analyses will help dissect the fine-scale kinetic mechanisms underlying *AhLOG*-mediated stress adaptation in peanut.

In summary, this study reveals that the peanut *AhLOG* gene family has undergone segmental duplication-driven lineage-specific expansion under strong purifying selection. While maintaining core catalytic structures, its members have evolved functional divergence closely related to peanut-specific developmental processes (pod enlargement, nodule symbiosis) and abiotic stress responses. The identified key candidate genes (e.g., *AhLOG3/16* highly expressed in pods, *AhLOG19* specific to nodules, *AhLOG2/5/6/9/10/11* responsive to dual stresses) provide clear targets for subsequent functional verification. In the future, combined with gene editing and transgenic technologies, it is necessary to further dissect the precise molecular mechanisms of these genes in regulating cytokinin homeostasis and coordinating growth, development, and stress resistance, so as to provide theoretical basis and genetic resources for high-yield and stress-resistant breeding of peanut.

## 4. Materials and Methods

### 4.1. Genome-Wide Identification of LOG Gene Family in Peanut

In this study, cultivated peanut (*Arachis hypogaea* L., AABB genome) and its diploid wild progenitors (*A. duranensis*, AA genome; *A. ipaensis*, BB genome), as well as *A. monticola*, were used as research materials. The genome sequences, protein sequences, and annotation files of cultivated peanut (*Arachis hypogaea cv.* Tifrunner, version 2.0, AABB, BioProject: PRJNA419393), as well as its two diploid progenitors *A. duranensis* (AA genome, BioProject: PRJNA316327) and *A. ipaensis* (BB genome, BioProject: PRJNA258025), used in this study were retrieved from the PeanutBase database (https://www.peanutbase.org/, accessed on 27 March 2026) [53]. The genomic data of *A. monticola* (AABB) was obtained from the National Genomics Data Center (NGDC) at the China National Center for Bioinformation (https://ngdc.cncb.ac.cn/, accessed on 27 March 2026, BioProject: PRJCA019219) [54].

To comprehensively identify the *LOG* (LONELY GUY) gene family, a combined strategy of hidden Markov model (HMM) search and BLASTp homology alignment was adopted. First, the HMM profile of the LOG conserved domain Lysine_decarbox (PF03641) [41,42] was downloaded from the Pfam database (http://pfam.xfam.org/, accessed on 28 March 2026), and HMMER software (v3.3.2) was used to search the peanut proteome with an E-value threshold of ≤10^−20^. Candidate hits were further filtered to retain only those with a LOG domain coverage >80% and a predicted protein length ≥90 amino acids. Meanwhile, known LOG protein sequences from *Arabidopsis thaliana* [41] and *Oryza sativa* [52] were used as queries to perform BLASTp against the peanut proteome, with thresholds of E-value < 10^−5^, sequence identity ≥ 40%, and alignment coverage ≥ 50%. Candidate sequences were further validated by reciprocal BLASTp alignment against the SwissProt database (UniProt release 2023_02). Conserved LOG domains were authenticated using the NCBI Conserved Domain Searchtool; sequences lacking complete LOG domains (coverage < 70%) or containing premature stop codons were excluded from further analysis. The intersection of candidate genes obtained by the two methods was taken as the initial candidate set.

The intersection of candidates from both methods was retained as the initial set and further validated using SMART online tool (https://smart.embl.de/, accessed on 1 April 2026) [55], keeping only sequences with an intact Lysine_decarbox domain. To remove splice variants and fragmented annotations, only the longest transcript per locus encoding a complete Lysine_decarbox (PF03641) domain was retained. Candidates with >95% identity at overlapping genomic coordinates were manually removed as isoforms, whereas highly similar sequences on distinct chromosomes were preserved based on phylogenetic and synteny evidence. Homologous *LOG* genes in wild *Arachis* species were retrieved using the same criteria.

### 4.2. Prediction of Physicochemical Properties and Subcellular Localization

The physicochemical properties of the identified AhLOG proteins, including amino acid length, theoretical molecular weight, isoelectric point (pI), instability index, aliphatic index, and grand average of hydropathicity (GRAVY), were analyzed using the ExPASy ProtParam web server (http://web.expasy.org/protparam/, accessed on 2 April 2026) [56]. Subcellular localization of AhLOG proteins was predicted using the WoLF PSORT web server (https://wolfpsort.hgc.jp/, accessed on 2 April 2026) [57], and the position with the highest confidence was selected as the final localization result.

### 4.3. Phylogenetic Analysis of the LOG Gene Family

LOG protein sequences from cultivated peanut, wild peanut, *A. thaliana*, and *O. sativa* were combined for multiple sequence alignment using the Clustal program integrated in MEGA 12.1.2 software [58]. A phylogenetic tree was constructed using the Maximum Likelihood (ML) method, with 1000 bootstrap replicates to evaluate branch confidence. The Newick-format tree file was imported into the iTOL online tool (https://itol.embl.de/, accessed on 3 April 2026) for visualization [59].

### 4.4. Chromosomal Localization and Gene Duplication Analysis of AhLOG Genes

Based on the peanut genome GFF annotation file, the Gene Location Visualize from GTF/GFF function in TBtools v2.458 was used to map *AhLOG* genes to chromosomes and complete visualization. *AhLOG* members were named according to their physical positions on the chromosomes [60].

Intraspecific collinearity of peanut *LOG* genes was analyzed using the One Step MCScanX tool in TBtools to identify segmental and tandem duplication events. The collinearity results were visualized using the Advanced Circos function [60].

### 4.5. Interspecific Synteny Analysis

Interspecific synteny analyses were performed separately between cultivated peanut and *A. duranensis*, *A. ipaensis*, *A. monticola*, and between peanut and *A. thaliana*, *O. sativa* using the One Step MCScanX tool [60]. The Multiple Synteny Plot tool was used to jointly visualize the multi-species synteny results, revealing the evolutionary conservation of the *LOG* gene family across species.

### 4.6. Analysis of Gene Structure and Conserved Motifs

Genome annotation files of peanut were downloaded from PeanutBase, and TBtools was used to extract the exon–intron and UTR structure information of *AhLOG* genes and draw gene structure maps. The full-length protein sequences of AhLOG were submitted to the MEME online tool (v5.5.9) for conserved motif analysis [56], with parameters set as follows: maximum number of motifs = 10, motif width = 6–50 aa. The MEME XML results were imported into TBtools to realize the integrated visualization of phylogenetic tree, gene structure, and conserved motifs.

### 4.7. Analysis of Cis-Acting Regulatory Elements in Promoters

The 2000 bp sequences upstream of the transcription start site of each *AhLOG* genes were extracted as candidate promoter regions. Cis-acting regulatory elements were predicted and annotated using the PlantCARE database (https://bioinformatics.psb.ugent.be/webtools/plantcare/html/, accessed on 4 April 2026) [61]. TBtools was used to visualize the type, number and distribution of cis-elements, and analyze the transcriptional regulatory characteristics of *AhLOG* genes.

### 4.8. Transcriptome-Based Expression Analysis of AhLOG Genes

Tissue-specific expression data of the *AhLOG* gene family were obtained from the public transcriptome dataset of *Arachis hypogaea* cv. Tifrunner (https://www.ncbi.nlm.nih.gov/, accessed on 27 March 2026, BioProject: PRJNA291488), covering 22 tissues/organs including roots, nodules, leaves, floral organs, pegs, pods, and seeds at different developmental stages [53]. FPKM (Fragments Per Kilobase of transcript per Million mapped reads) values were used to assess gene expression levels, and low-expression genes (FPKM < 0.1 in all tissues) were filtered out.

### 4.9. qRT-PCR Expression Analysis of AhLOG Genes Under Hormone and Abiotic Stress Treatments

Peanut seedlings at the three-leaf stage were used for hormone and abiotic stress treatments. For hormone treatments, seedlings were treated with 20 μM abscisic acid (ABA), 100 μM gibberellin (GA), 100 μM indole-3-acetic acid (IAA), and 10 μM methyl jasmonate (MeJA), respectively, and leaf tissues were harvested at 0, 1, 3, 5, 7, and 9 h after treatment. For abiotic stress treatments, seedlings were exposed to 200 mM NaCl (salt stress) and 350 mM PEG 6000 (simulated drought stress), and root tissues were collected at 0, 2, 4, 6, 8, 10, 12, and 24 h after treatment. As cultivated peanut is an allotetraploid (AABB) with high sequence similarity between the A and B subgenomes, qRT-PCR was performed to detect the expression levels of 12 *AhLOG* genes (*AhLOG1–12*) located on the A subgenome.

Total RNA was extracted from young leaf tissues using the TaKaRa MiniBEST Plant RNA Extraction Kit (TaKaRa Bio Inc., Kusatsu, Shiga, Japan) according to the manufacturer’s instructions. DNase was applied to eliminate genomic DNA contamination during RNA purification, and total RNA was finally eluted with RNase-free water. RNA degradation and contamination were preliminarily detected by 1% agarose gel electrophoresis, and RNA quality was further verified using the 2100 Bioanalyzer (Agilent Technologies, Santa Clara, CA, USA). All qualified RNA samples met the criteria: OD_260_/OD_280_ ≥ 1.8 and 28S/18S ≈ 2. cDNA was synthesized from the purified total RNA using the PrimeScript™ II 1st Strand cDNA Synthesis Kit (TaKaRa, Dalian, China). qRT-PCR primers for *AhLOG* genes were designed from their CDSs using Primer3, with specificity verified by BLAST (https://www.ncbi.nlm.nih.gov/tools/primer-blast/, accessed on 4 April 2026) against the peanut genome (Table S5). qRT-PCR was performed using the Power Up™ SYBR™ Green Master Mix (Applied Biosystems Inc., Irvine, CA, USA). *AhADH3* was selected as the internal reference gene for normalization [47]. Each sample contained three biological replicates and three technical replicates. The relative expression levels of *AhLOG* genes were calculated using the 2^−ΔΔCt^ method (Table S4). Student’s *t*-test was performed via GraphPad Prism 8 software to identify expression differences between each treatment time point and the 0 h untreated control, with significance thresholds set at * *p* < 0.05 and ** *p* < 0.01.

## 5. Conclusions

This study presents the first systematic genome-wide characterization of the *AhLOG* gene family in cultivated peanut, revealing its evolutionary conservation, segmental duplication-driven expansion, tissue-specific expression divergence, and functional differentiation in development and abiotic stress responses. *AhLOG3* and *AhLOG16* are identified as core candidates involved in growth, development, and stress tolerance, supported by their conserved structures, canonical LOG domains, and broad expression patterns. qRT-PCR analysis further showed that most *AhLOG* genes are significantly induced by ABA, GA, IAA, MeJA, drought, and salt stresses, with six members simultaneously responding to both osmotic and ionic stresses. Other *AhLOG* members exhibit strict tissue-specific expression (e.g., roots, leaves, floral organs, nodules, pods), suggesting specialized functions in peanut-specific developmental processes. Subcellular localization prediction indicates that most AhLOG proteins are targeted to the cytoplasm, consistent with their role in cytokinin activation. Collectively, these findings provide a solid foundation for functional validation of the *AhLOG* gene family and offer valuable genetic targets for molecular breeding to improve abiotic stress resilience and yield stability in peanut. Future studies should employ subgenome-specific primers or homeolog-resolved transcriptomic approaches to compare A and B subgenome *AhLOG* expression under stress conditions, thereby testing the hypothesized regulatory divergence.

## Figures and Tables

**Table 1:** The Physicochemical characterization of *AhLOG* gene family members in *Arachis hypogaea* cv. Tifrunner.

Chr	Gene ID	Gene Name	Protein Length (aa)	Molecular Weight (MW)/Da	Isoelectric Point (pi)	Subcellular Localization
Chr01	AhLOG1	Ah01g027700.1	316	34,862.43	40.19	Chloroplast
Chr02	AhLOG2	Ah02g388800.1	213	23,336.76	42.56	Cytoplasm
Chr02	AhLOG3	Ah02g407600.1	219	23,957.46	39.15	Cytoplasm
Chr03	AhLOG4	Ah03g012800.1	124	14,501.12	54.95	Endoplasmic reticulum
Chr03	AhLOG5	Ah03g017600.1	231	25,476.87	55.27	Cytoplasm
Chr03	AhLOG6	Ah03g020800.1	91	10,131.76	47.86	Cytoplasm
Chr03	AhLOG7	Ah03g114800.1	226	24,649.32	36.77	Cytoplasm
Chr06	AhLOG8	Ah06g159500.1	215	23,517.27	38.97	Cytoplasm
Chr06	AhLOG9	Ah06g191800.1	220	24,271.84	37.91	Cytoplasm
Chr08	AhLOG10	Ah08g018400.1	223	24,274.91	40.60	Cytoplasm
Chr08	AhLOG11	Ah08g115900.1	216	23,555.99	36.09	Cytoplasm
Chr08	AhLOG12	Ah08g176700.1	211	23,197.67	38.98	Cytoplasm
Chr11	AhLOG13	Ah11g079100.1	320	35,405.02	43.32	Chloroplast
Chr11	AhLOG14	Ah11g174800.1	92	10,556.20	28.66	Cytoplasm
Chr12	AhLOG15	Ah12g456900.1	213	23,336.76	42.56	Cytoplasm
Chr12	AhLOG16	Ah12g475800.1	219	23,957.46	39.15	Cytoplasm
Chr13	AhLOG17	Ah13g037000.1	233	25,776.19	59.65	Cytoplasm
Chr13	AhLOG18	Ah13g147600.1	226	24,661.36	36.93	Cytoplasm
Chr16	AhLOG19	Ah16g009000.1	219	24,054.80	39.63	Cytoplasm
Chr16	AhLOG20	Ah16g228500.1	220	24,271.84	37.91	Cytoplasm
Chr16	AhLOG21	Ah16g315000.1	123	13,865.13	71.23	Nucleus
Chr17	AhLOG22	Ah17g358800.1	223	24,274.91	40.60	Cytoplasm
Chr17	AhLOG23	Ah17g488500.1	216	23,611.07	37.60	Cytoplasm
Chr18	AhLOG24	Ah18g057100.1	211	23,196.69	41.65	Cytoplasm

**Table 2:** Intraspecific gene pairs and Ka/Ks analysis in *Arachis hypogaea.*

Gene Name	Gene Name	Ka	Ks	Ka/Ks
*AhLOG1*	*AhLOG13*	0.01389	0.03229	0.43027
*AhLOG3*	*AhLOG9*	0.02830	0.47810	0.05919
*AhLOG3*	*AhLOG12*	0.09012	0.80915	0.11137
*AhLOG2*	*AhLOG21*	0.22129	1.12740	0.19629
*AhLOG3*	*AhLOG20*	0.02831	0.45294	0.06249
*AhLOG3*	*AhLOG24*	0.08777	0.83014	0.10572
*AhLOG6*	*AhLOG12*	0.29186	0.81406	0.35853
*AhLOG5*	*AhLOG12*	0.07037	0.45484	0.15471
*AhLOG7*	*AhLOG18*	0.00769	0.02622	0.29336
*AhLOG5*	*AhLOG17*	0.00561	0.03272	0.17143
*AhLOG6*	*AhLOG17*	0.20837	0.41693	0.49978
*AhLOG4*	*AhLOG17*	0.49661	0.63369	0.78367
*AhLOG6*	*AhLOG24*	0.29186	0.81406	0.35853
*AhLOG5*	*AhLOG24*	0.06812	0.45484	0.14977
*AhLOG8*	*AhLOG10*	0.06534	0.89123	0.07331
*AhLOG9*	*AhLOG16*	0.02830	0.47810	0.05919
*AhLOG9*	*AhLOG20*	0.00000	0.04076	0.00000
*AhLOG8*	*AhLOG19*	0.00403	0.01364	0.29589
*AhLOG8*	*AhLOG22*	0.06534	0.93645	0.06977
*AhLOG12*	*AhLOG17*	0.07432	0.46175	0.16096
*AhLOG10*	*AhLOG19*	0.06312	0.87144	0.07243
*AhLOG11*	*AhLOG23*	0.00200	0.00676	0.29636
*AhLOG10*	*AhLOG22*	0.00000	0.01929	0.00000
*AhLOG12*	*AhLOG24*	0.00205	0.00700	0.29219
*AhLOG15*	*AhLOG21*	0.22129	1.12740	0.19629
*AhLOG16*	*AhLOG20*	0.02831	0.45294	0.06249
*AhLOG17*	*AhLOG24*	0.07207	0.46175	0.15609
*AhLOG19*	*AhLOG22*	0.06312	0.91549	0.06894

## Data Availability

The genome assembly, genome sequence, annotation, coding sequence (CDS), and protein sequence files of *Arachis hypogaea* cv. Tifrunner used in this study were retrieved from PeanutBase (https://www.peanutbase.org/, accessed on 27 March 2026), including genome assembly arahy.Tifrunner.gnm2.J5K5 (NCBI Assembly: GCF_003086295.3), genome sequence file arahy.Tifrunner.gnm2.J5K5.genome_main.fna, annotation file arahy.Tifrunner.gnm2.ann2.PVFB.gene_models_main.gff3, CDS file arahy.Tifrunner.gnm2.ann2.PVFB.cds.fna, and protein sequence file arahy.Tifrunner.gnm2.ann2.PVFB.protein.faa. Genome sequences of *A. duranensis* and *A. ipaensis* were obtained from PeanutBase (https://www.peanutbase.org/, accessed on 27 March 2026). Genomic data of *A. monticola* were acquired from the National Genomics Data Center (https://ngdc.cncb.ac.cn/, accessed on 27 March 2026, Accession: PRJCA019219). The hidden Markov model (HMM) profile of the LOG domain (PF03641) was downloaded from the Pfam database (http://pfam.xfam.org/, accessed on 28 March 2026). The public transcriptome dataset covering 22 tissues of *A. hypogaea* cv. Tifrunner was obtained from the National Center for Biotechnology Information (NCBI, Accession: PRJNA291488). All gene identifiers, Ka/Ks calculations, cis-element counts, qRT-PCR primer sequence and raw qRT-PCR quantification data are provided in Supplementary Tables S1–S5. Further inquiries can be directed to the corresponding authors.

## Supplementary Materials

The following supporting information can be downloaded at: https://www.mdpi.com/article/10.3390/ijms27114958/s1.
